# Supplementary material for: Surface Microbiome Profiling of Dental Elevators Using Third‐Generation Sequencing: Implications for Infection Control in Dental Practice
Source: Microbiologyopen. 2025 Dec 8;14(6):e70178. doi: 10.1002/mbo3.70178 (PMC12685768; doi:10.1002/mbo3.70178)
Supplement: Supplementary file 1 — customer_backup. [file MBO3-14-e70178-s001.zip › customer_backup/customer_backup/taxa_summary/heatmap/treat/treat.order.reabundance.heatmap.pdf]

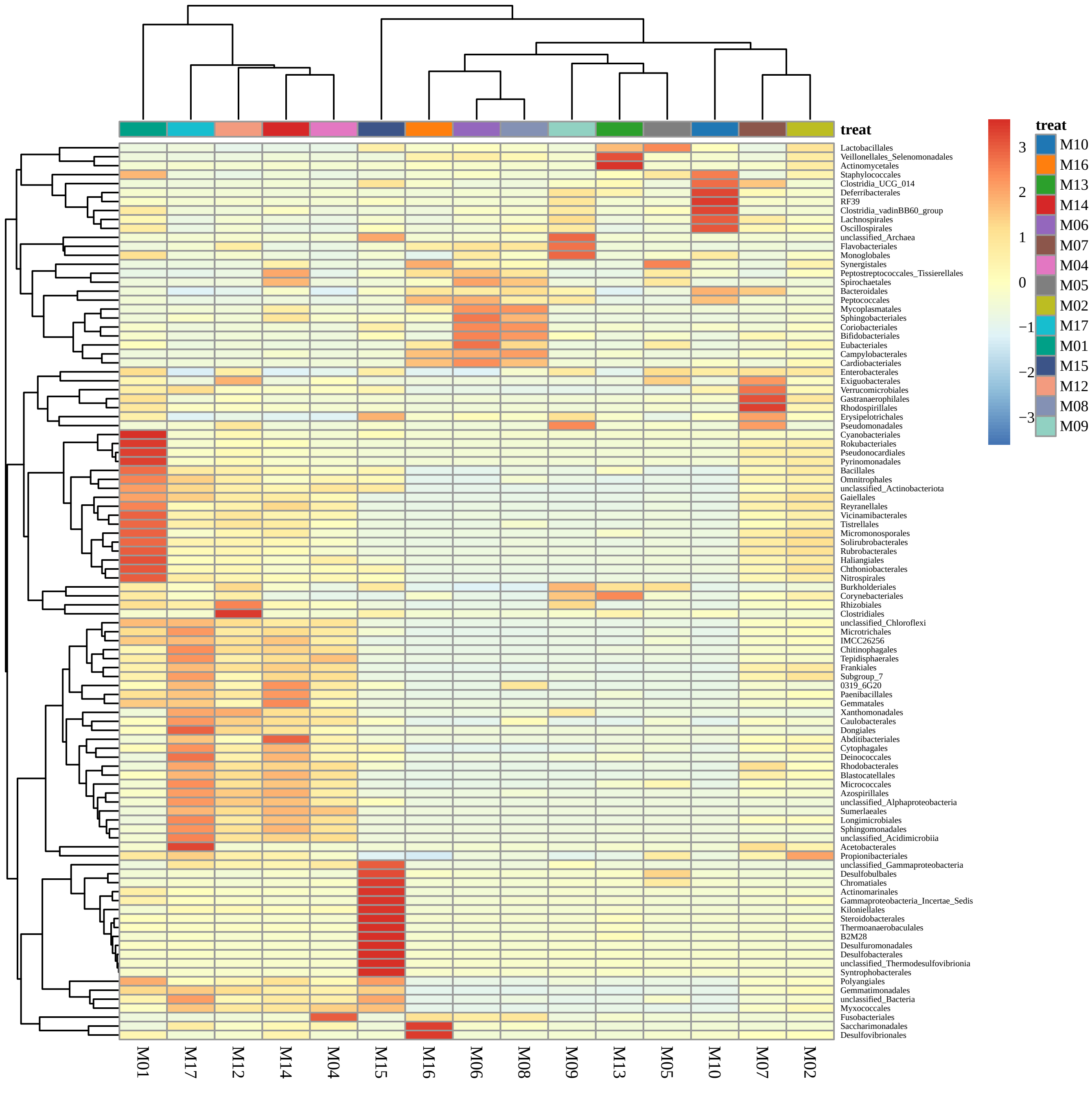
treat
Lactobacillales
Veillonellales_Selenomonadales
Actinomycetales
Staphylococcales
Clostridia_UCG_014
Deferribacterales
RF39
Clostridia_vadinBB60_group
Lachnospirales
Oscillospirales
unclassified_Archaea
Flavobacteriales
Monoglobales
Synergistales
Peptostreptococcales_Tissierellales
Spirochaetales
Bacteroidales
Peptococcales
Mycoplasmatales
Sphingobacteriales
Coriobacteriales
Bifidobacteriales
Eubacteriales
Campylobacterales
Cardiobacteriales
Enterobacterales
Exiguobacterales
Verrucomicrobiales
Gastranaerophilales
Rhodospirillales
Erysipelotrichales
Pseudomonadales
Cyanobacteriales
Rokubacteriales
Pseudonocardiales
Pyrinomonadales
Bacillales
Omnitrophales
unclassified_Actinobacteriota
Gaiellales
Reyranellales
Vicinamibacterales
Tistrellales
Micromonosporales
Solirubrobacterales
Rubrobacterales
Haliangiales
Chthoniobacterales
Nitrospirales
Burkholderiales
Corynebacteriales
Rhizobiales
Clostridiales
unclassified_Chloroflexi
Microtrichales
IMCC26256
Chitinophagales
Tepidisphaerales
Frankiales
Subgroup_7
0319_6G20
Paenibacillales
Gemmatales
Xanthomonadales
Caulobacterales
Dongiales
Abditibacteriales
Cytophagales
Deinococcales
Rhodobacterales
Blastocatellales
Micrococcales
Azospirillales
unclassified_Alphaproteobacteria
Sumerlaeales
Longimicrobiales
Sphingomonadales
unclassified_Acidimicrobiia
Acetobacterales
Propionibacteriales
unclassified_Gammaproteobacteria
Desulfobulbales
Chromatiales
Actinomarinales
Gammaproteobacteria_Incertae_Sedis
Kiloniellales
Steroidobacterales
Thermoanaerobaculales
B2M28
Desulfuromonadales
Desulfobacterales
unclassified_Thermodesulfovibrionia
Syntrophobacterales
Polyangiales
Gemmatimonadales
unclassified_Bacteria
Myxococcales
Fusobacteriales
Saccharimonadales
Desulfovibrionales
M01
M17
M12
M14
M04
M15
M16
M06
M08
M09
M13
M05
M10
M07
M02
3
2
1
0
−1
−2
−3
treat
M10
M16
M13
M14
M06
M07
M04
M05
M02
M17
M01
M15
M12
M08
M09
